# Impact of patient gender on surgical outcomes of infective endocarditis in adults: a systematic review and meta-analysis protocol

**DOI:** 10.1093/jsprm/snaf004

**Published:** 2025-05-13

**Authors:** Fay Fathima Imtiaz Fareed, Niraj S Kumar, Ruhani Singh, Wael I Awad

**Affiliations:** University of Buckingham Medical School, Buckingham MK18 1EG, UK; National Medical Research Association, London, UK; National Medical Research Association, London, UK; Department of Cardiovascular Sciences, University of Leicester, University Road, Leicester LE1 7RH, UK; National Medical Research Association, London, UK; Barts Heart Centre, St. Bartholomew’s Hospital, London, UK; Centre for Cardiovascular Medicine and Devices, William Harvey Research Institute, Queen Mary University of London, London, UK; University of South Wales, Cardiff, UK

**Keywords:** cardiac surgery, infective endocarditis, gender, sex, outcomes

## Abstract

Introduction: Infective endocarditis is a rare but severe disease affecting 3–10 per 100 000 each year associated with a mortality of 25%. However, it is thought to affect females more severely than men, despite lower incidence. However, reasons for this are unknown, and there is controversy surrounding evidence for differences in surgical outcomes for infective endocarditis. Thus, a systematic review and meta-analysis is warranted to elucidate differences in outcomes by gender. Methods and analysis: This systematic review protocol has been developed in accordance with Preferred Reporting Items for Systematic Review and Meta-Analysis Protocols guidelines. A systematic search including synonyms of the terms ‘infective endocarditis’, ‘cardiac surgery’ and ‘sex’, was carried on MEDLINE, Embase and Scopus databases (full search available in the Supplementary Material) to identify relevant studies. The Cochrane Risk of Bias 2 tool and Newcastle-Ottawa Scale will be used to assess the quality of the available studies and risk of bias. Studies will be screened using predetermined inclusion and exclusion criteria. Data will be summarized narratively and in tabular forms. A pairwise meta-analysis will be carried out with a random effects model to examine differences in mortality and postoperative complications between males and females. Discussion: The findings will elucidate the influence of gender on surgical outcomes for infective endocarditis, informing evidence-based interventions and emphasizing the need for equitable surgical care. By identifying risk factors specific to women, this study aims to improve management strategies and outcomes for female patients with infective endocarditis. Results will be disseminated via peer-reviewed publications and relevant conferences.

## INTRODUCTION

Infective endocarditis (IE) is an acute, severe and life-threatening condition with an annual incidence of ~3–10 cases per 100 000 people and overall mortality of 25% globally [1]. Men are more commonly affected than women, with 50 cases per million inhabitants among men, compared to only 16 cases per million among women [2]. Possible reasons may be due to men being at increased risk of developing cardiac conditions or due to underdiagnosis of IE in women [3]. However, even within incidence, there is controversy with some studies reporting no difference between the genders [4].

Standards indications for surgical management of infective endocarditis include severe heart failure, severe valve dysfunction, prosthetic valve infection, invasion beyond valve, recurrent systemic embolization, large mobile vegetations or persistent sepsis despite treatment with antibiotics for more than 5–7 days [5]. Studies have shown that women undergo cardiac surgery for IE less frequently than men [6, 7]. Female sex is associated with increased mortality, especially in cases involving surgical management, and this is closely linked to the severity of the initial clinical presentation and increased age at presentation [2, 7]. The higher incidence of comorbidities such as diabetes mellitus and renal failure in women may also be a contributing factor to the poorer outcomes as compared to men [8]. Additionally, female patients have a higher incidence of postoperative heart failure, independent of the underlying pathology [9]. *Staphylococcus aureus*, the most common pathogen in IE, was also more commonly found in women than men and has been linked to higher rates of stroke, systemic emboli, persistent bacteremia and mortality [2, 10]. Long-term survival may be poorer in women, although current evidence shows contrasting results on this indicating the need for an up-to-date systematic review inclusive of more recent studies to resolve this discrepancy [2, 6].

When considering risk factors for developing IE, females have a higher prevalence of risks related to older age, mitral valve involvement and haemodialysis, but males have an increased prevalence of risks such as previous prosthetic valve replacement, periannular complications, coronary artery disease and liver cirrhosis [3, 5]. In view of this, the 2023 European Society of Cardiology guidelines further stressed the importance of addressing the impact of gender on outcomes in surgically treated patients [3]. Subsequently, this systematic review and meta-analysis aims to meticulously examine the existing literature, consolidate the findings and provide an in-depth analysis of the impact of patient gender on surgical outcomes in IE.

## MATERIALS AND METHODS

This systematic review is reported in adherence to the Preferred Reporting Items for Systematic Review and Meta-Analysis Protocols (PRISMA-P) recommendations [11].

The protocol has been registered with PROSPERO and the registration number is CRD42023484225. Any significant differences or changes to the protocol will be highlighted in the final manuscript.

### Review question

Our primary review question is to investigate the impact of patient gender on outcomes following cardiac surgery for infective endocarditis, especially focusing on mortality (in-hospital and long-term), length of stay, acute kidney injury, in-hospital stroke and sepsis.

### Search strategy

MEDLINE, EMBASE and Scopus databases will be searched from August 2023, and the search will be re-run during the screening process to ensure that the most recent studies available will be included. The searches were restricted to peer-reviewed articles written in English only. All relevant synonyms of ‘infective endocarditis’, ‘cardiac surgery’ and ‘gender’ will be used to develop the search strategy to ensure relevant articles are being examined (the full search strategy is available in Supplement Material S1). Additionally, we will search through the references of relevant identified systematic reviews and meta-analyses for additional papers for inclusion in our review.

### Inclusion and exclusion criteria

The identified papers will be assessed against predetermined inclusion and exclusion criteria. All included texts will need to be original research that studies patients undergoing cardiac surgical management for infective endocarditis. We will include papers based on any country and clinical setting. We will include both observational studies and trials, and exclude protocols, conference abstracts and non-journal articles.

### Assessment and screening

The Population, Intervention, Comparator, Outcomes, Study Design (PICOS) framework was used to aid in the assessment of the included studies (Table 1) [12].

**Table 1:** PICOS framework applied to this systematic review.

Population	Adult patients (>18 years) undergoing cardiac surgery for management of infective endocarditis
Intervention	Female patients undergoing cardiac surgery for management of infective endocarditis
Comparator	Male patients undergoing cardiac surgery for management of infective endocarditis
Outcomes	Mortality (in-hospital and long-term), length of stay, acute kidney injury, in-hospital stroke and sepsis
Study designs	Observational studies and randomized controlled trials

The number of results identified from the initial search will be reported. The studies will be input into the Rayyan tool for deduplication and further screening according to appropriate inclusion and exclusion criteria [13]. Two reviewers will be assigned to screen the titles and abstracts of all the studies independently, with a third reviewer being assigned to resolve conflicts. Full-text screening and data synthesis will follow a similar framework, with two reviewers assigned for full-text screening, and a third reviewer for conflict resolution.

### Quality assessment and risk of bias

The Cochrane Risk of Bias 2 (ROB2) tool will be utilized for the risk of bias assessment of included randomized controlled trials, which will be determined by assessing if there is a lack of allocation concealment, lack of blinding, incomplete accounting of outcome events, selective outcome reporting and/or other limitations [14].

The Newcastle-Ottawa Scale will be used for risk of bias assessment in observational studies, based on three parameters: selection, comparability between exposed and unexposed groups and assessment of outcome [15].

Two independent investigators will perform quality assessments, with disagreements resolved through a third independent reviewer. Quality scores will be reflected in the data synthesis results and will be reported, but no study will be excluded based on quality assessment.

The Grading of Recommendations Assessment, Development and Evaluation tool will be used to determine the certainty in evidence for each outcome [16]. It will assess limitations in study design, indirectness, imprecision, inconsistency and publication bias. Studies will be subsequently classified as either high, moderate, low, or very low.

### Data extraction

Data will be extracted and collected independently through Microsoft Excel (Microsoft Corporation, Redwood, VA) after full-text screening. Extraction will be completed by two authors, with any disagreements being resolved via discussion.

All data will be summarized in tabular forms and will be further described in the results section. Two authors will independently extract the following data: study design, study population and outcome measures using the prespecified tables. This will be extracted and reported in the systematic review to ensure adequate comparison of patients within included studies and to ascertain whether these factors may influence surgical outcomes. Data to be extracted includes:

1. Study characteristics (First author, Year, Journal, Study Design, Type of surgery, Sample Size, Comparator, Location, Other clinical information collected, Risk of Bias Score, Quality of Evidence Score),
2. Population characteristics: mean age (years ± SD), sex (M/F), hypertension (%), diabetes mellitus (%), chronic obstructive pulmonary disease (%), myocardial Infarction (%), heart failure (%), ejection fraction (%), BMI (kg/m^2^), CRF (%), NYHA Class II/III, renal function (chronic kidney disease status), smoking status, peripheral vascular disease (%).
3. Operative characteristics: type of surgery (coronary artery bypass graft, valve repair/replacement, aortic surgery, other adult cardiac operations), operative approach and anaesthesia regimen
4. Outcomes: mortality (in-hospital and long-term), length of stay, acute kidney injury, in-hospital stroke, sepsis and conduction abnormalities requiring permanent pacemaker implantation.

### Data synthesis

For all variables for which there is sufficient sample size a random effects meta-analysis will be conducted using Mantel–Haenszel method for all outcome variables to determine a pooled odd’s ratio and 95% confidence intervals, will be performed with the RevMan 5.4.1 tool (Nordic Cochrane Centre, Copenhagen, Denmark). The *I*^2^ statistic will be used to assess the consistency among studies in accordance with guidance from the *Cochrane Handbook* [17]. Results with a *P*-value <0.05 will be considered statistically significant. We will produce Forest plots to illustrate the meta-analyses. Additionally, funnel plots will be generated to assess for publication bias and for any outcomes reported in over 10 studies, Egger’s test will be conducted.

### Subgroup analysis

Where possible and sufficiently reported in at least two included studies, we will conduct subgroup analysis to determine the effect of comorbidities on surgical outcomes. Potential analyses may be conducted by study design, participants characteristics, and by the cardiac surgery procedure undergone by the patient (e.g. aortic valve replacement).

## DISCUSSION

This systematic review is the first to examine the impact of gender differences on surgical outcomes for infective endocarditis. The evidence gathered will be used to elucidate the influence of gender on outcomes such as mortality (in-hospital and long-term), length of stay, acute kidney injury, in-hospital stroke, sepsis and conduction abnormalities requiring permanent pacemaker implantation.

Emerging evidence underscores the multifactorial nature of gender disparities in outcomes following cardiac surgery for infective endocarditis. For instance, studies have demonstrated that female patients often present with a greater burden of comorbidities—such as renal dysfunction and diabetes mellitus—that predispose them to heightened postoperative complications [4, 6]. Moreover, female patients have disproportionate prevalence of *S. aureus* infections, a risk factor associated with increased risks of systemic embolization and persistent bacteraemia, which have been associated with risk of sepsis and postoperative mortality [2, 10]. These findings, together with observations regarding potential delays in diagnosis and surgical intervention among female patients, suggest that both inherent biological differences and variations in clinical management may contribute to the observed outcome disparities [6–8].

Our findings, by synthesizing and clarifying these insights and examining outcome data, hope to encourage the implementation of effective risk mitigation and consideration for early surgical intervention for female patients to prevent morbidity and mortality. A subsequent meta-analysis of all included studies will allow for pooling of results and provide a comparison of outcomes between genders with greater power. Further, this study intends to promote evidence-based surgical decision-making when considering surgical management for patients with infective endocarditis.

## STRENGTHS AND LIMITATIONS

Strengths of this review will include using a comprehensive search strategy developed after consulting experts in the field, following the PRISMA-P recommendations and a systematic review process by two independent authors that will ensure adequate coverage of all the available data at this time. The risk of bias will be assessed appropriately using the Newcastle-Ottawa Scale for observational studies and the Cochrane Risk of Bias 2 tool for randomized controlled trials.

Limitations include the potential bias in establishing inclusion and exclusion criteria, which can restrict the applicability of the findings to specific populations or interventions. Finally, only studies in the English language were included. This was decided due to language constraints within the team, hindering the usage of studies in other languages at this stage.

## ETHICS AND DISSEMINATION

This systematic review does not involve patient data and, therefore, does not require ethical approval. Findings will be disseminated through publications in peer-reviewed journals and presentations at relevant conferences. The results aim to influence clinical guidelines and practices, promoting equity in surgical outcomes for IE patients.

## Supplementary Material

S1_PRISMA-P_checklist-Protocol_IE_snaf004

S2_Protocol_Search_Strategy_snaf004

## Data Availability

Data sharing is not applicable to this article as no datasets will be generated or analysed during the current study.

## PATIENT AND PUBLIC INVOLVEMENT

Patients and/or the public were not involved in the design, conduct, reporting, or dissemination plans for this research.
