# Supplementary material for: Impact of patient gender on surgical outcomes of infective endocarditis in adults: a systematic review and meta-analysis protocol
Source: J Surg Protoc Res Methodol. 2025 May 13;2025:snaf004. doi: 10.1093/jsprm/snaf004 (PMC12080235; doi:10.1093/jsprm/snaf004)
Supplement: S2_Protocol_Search_Strategy_snaf004 [file s2_protocol_search_strategy_snaf004.docx]

Supplement 1: Search strategy

Medline (via OVID)

1. Endocarditis, Bacterial/

2. (infective endocarditis or bacterial endocarditis or bacterial heart infection or IE).tw,kf.

3. Cardiac Surgical Procedures/

4. (cardiac surgery or heart surgery or cardiovascular surgery or cardiac operation or heart operation or cardiovascular operation or cardiac procedures or heart procedures or valve surgery or valve replacement).tw,kf.

5. Sex Factors/

6. (sex or gender or male or female or men or women or sex characteristics or gender differences or sex differences).tw,kf.

7. 1 or 2

8. 3 or 4

9. 5 or 6

10. 7 and 8 and 9

11. limit 10 to (humans and english language)

Embase (via OVID)

1. 'infective endocarditis'/exp

2. (infective endocarditis or bacterial endocarditis or bacterial heart infection or IE):ti,ab,kw

3. 'heart surgery'/exp

4. (cardiac surgery or heart surgery or cardiovascular surgery or cardiac operation or heart operation or cardiovascular operation or cardiac procedures or heart procedures or valve surgery or valve replacement):ti,ab,kw

5. 'sex difference'/exp

6. (sex or gender or male or female or men or women or sex characteristics or gender differences or sex differences):ti,ab,kw

7. 1 or 2

8. 3 or 4

9. 5 or 6

10. 7 and 8 and 9

11. limit 10 to (human and english language)

Scopus

(TITLE-ABS-KEY("infective endocarditis") OR TITLE-ABS-KEY("bacterial endocarditis") OR TITLE-ABS-KEY("bacterial heart infection") OR TITLE-ABS-KEY("IE"))

AND (TITLE-ABS-KEY("cardiac surgery") OR TITLE-ABS-KEY("heart surgery") OR TITLE-ABS-KEY("cardiovascular surgery") OR TITLE-ABS-KEY("cardiac operation") OR TITLE-ABS-KEY("heart operation") OR TITLE-ABS-KEY("cardiovascular operation") OR TITLE-ABS-KEY("cardiac procedures") OR TITLE-ABS-KEY("heart procedures") OR TITLE-ABS-KEY("valve surgery") OR TITLE-ABS-KEY("valve replacement"))

AND (TITLE-ABS-KEY("sex") OR TITLE-ABS-KEY("gender") OR TITLE-ABS-KEY("male") OR TITLE-ABS-KEY("female") OR TITLE-ABS-KEY("men") OR TITLE-ABS-KEY("women") OR TITLE-ABS-KEY("sex characteristics") OR TITLE-ABS-KEY("gender differences") OR TITLE-ABS-KEY("sex differences"))

AND (LIMIT-TO(LANGUAGE, "English"))

AND (LIMIT-TO(DOCTYPE, "ar"))
